# Application of Metabolomics in Diagnosis of Cow Mastitis: A Review

**DOI:** 10.3389/fvets.2021.747519

**Published:** 2021-10-08

**Authors:** Honghong Hu, Zhou Fang, Tong Mu, Zhong Wang, Yun Ma, Yanfen Ma

**Affiliations:** Ningxia Key Laboratory of Ruminant Molecular and Cellular Breeding, School of Agriculture, Ningxia University, Yinchuan, China

**Keywords:** subclinical mastitis, clinical mastitis, metabonomics, diagnostic technique, omics technology

## Abstract

Cow mastitis, with high incidence rate and complex cause of disease, is one of the main diseases that affect the development of dairy industry in the world. Clinical mastitis and subclinical mastitis caused by *Staphylococcus aureus, Escherichia coli, Streptococcus*, and other pathogens have a huge potential safety hazard to food safety and the rapid development of animal husbandry. The economic loss caused by cow mastitis is billions of dollars every year in the world. In recent years, the omics technology has been widely used in animal husbandry with the continuous breakthrough of sequencing technology and the continuous reduction of sequencing cost. For dairy cow mastitis, the traditional diagnostic technique, such as histopathological screening, somatic cell count, milk pH test, milk conductivity test, enzyme activity test, and infrared thermography, are difficult to fully and comprehensively clarify its pathogenesis due to their own limitations. Metabolomics technology is an important part of system biology, which can simultaneously analyze all low molecular weight metabolites such as amino acids, lipids, carbohydrates under the action of complex factors including internal and external environment and in a specific physiological period accurately and efficiently, and then clarify the related metabolic pathways. Metabolomics, as the most downstream of gene expression, can amplify the small changes of gene and protein expression at the level of metabolites, which can more fully reflect the cell function. The application of metabolomics technology in cow mastitis can analyze the hetero metabolites, identify the related biomarkers, and reveal the physiological and pathological changes of cow mammary gland, so as to provide valuable reference for the prediction, diagnosis, and treatment of mastitis. The research progress of metabolomics technology in cow mastitis in recent years was reviewed, in order to provide guidance for the development of cow health and dairy industry safety in this manuscript.

## Introduction

Dairy cow mastitis is one of the diseases with the highest incidence in the world (1), and it is a key factor restricting the health of dairy cows and the safety of dairy products. Dairy cow mastitis is an inflammatory reaction of mammary gland tissue, which is induced by many factors, such as microbial infection (bacteria, fungi, mycoplasma, virus, etc.), environmental factors (sanitary conditions, temperature and humidity, feed, etc.), human factors (mechanical injury, milking stress, improper feeding management, etc.) and cow's own factors (such as age, parity, feed, milk yield, lactation stage, etc.) (2, 3).

Mastitis is characterized by mild or severe pathological changes in udder tissue, increased somatic cells count (SCC) and abnormal milk quality (4). Dairy cow mastitis is the most expensive infectious disease in dairy cow breeding worldwide. In terms of the impact on animal husbandry in the United States alone, the annual losses caused by the decline of milk production and quality, the soaring cost of veterinary treatment and the increasing cost of farm management are as high as billions of dollars (5). Cow mastitis is usually divided into clinical mastitis and subclinical mastitis according to the visible changes of udder and milk (6), and there are 137 kinds of pathogenic bacteria in dairy cow mastitis (7, 8), and *Staphylococcus aureus, Escherichia coli, Streptococcus* are the most common pathogens that cause mastitis (9, 10).

There are a lot of researches focused on genomics (11), transcriptomics (12), proteomics (13), and epiomics (14) in dairy cow mastitis. However, metabolomics, as the most downstream of gene expression, is gradually used to analyze the occurrence of dairy cow mastitis because it can reflect the current situation of biological systems, and it is relatively cheaper to implement (15). Traditional mastitis detection methods are difficult to fully and comprehensively elucidate its pathogenesis due to its time-consuming, low sensitivity, low specificity and accuracy, and other limitations (16, 17), but these methods can be relatively cheap. The emergence of metabolomics technology can help researchers to find the different changes of metabolites between healthy cows and cows with different degrees of inflammation, and screen out the effective biomarkers for timely and accurate prevention (18). Additionally, metabolomics can provide accurate targets for the development of therapeutic drugs for mastitis, and ultimately effectively prevent and control the onset of mastitis, the cost is also relatively high. To sum up, the research progress of metabolomics technology in the field of cow mastitis is reviewed, in order to provide more valuable information for the early diagnosis of cow mastitis, and provided new ideas for the follow-up research of cow health and dairy safety in this manuscript.

## Dairy Cow Mastitis

Dairy cow mammary gland is affected by many factors, including environment, feeding management, age, parity, pathogenic fungi, bacteria, mycoplasma, viruses, and other factors (3, 19). Once the pathogen invades the udder, it will produce a corresponding immune response to avoid infection.

### Current Situation of Cow Mastitis

Cow mastitis is divided into subclinical mastitis and clinical mastitis according to the change of SCC and metabolites in mastitis milk (20). There is few visual index and SCC (<50 × 10^4^/mL) in milk or udder of subclinical mastitis (21, 22), therefore, milk from subclinical mastitis is more likely to enter the milk bulk tank without knowing it. In most cases, cow mastitis is a chronic disease with subclinical or mild inflammation, subclinical mastitis will eventually develop into clinical mastitis if it is not treated in time (23). Clinical mastitis can be found by veterinarians timely (21). Clinical mastitis is characterized by increased SCC (>50 × 10^4^/mL) in the milk and the presence of visual signs of inflammation, such as clots, watery milk, blood like milk, udder swelling, tenderness, and so on. In addition, pathogens may be found in clinical mastitis milk (24). More than 100 pathogenic microorganisms cause cow mastitis (25, 26), *Staphylococcus aureus, Streptococcus, Escherichia coli* are the common athogenic microorganisms (27). Mastitis cannot be detected and is time-consuming when using bacterial detection methods to diagnose small sample populations. Bacteria must grow until their phenotype can be recognized before they can be detected, so it will take at least a few days before accurate results can be obtained (28). Therefore, looking for markers of subclinical mastitis will help us to detect diseased cows as soon as possible and to prevent and treat them.

As we all know, SCC and bacteria detection are the main methods for diagnosis of clinical mastitis. Although both methods have shortcomings, they are still widely used in the diagnosis of cow mastitis. SCC has been proved to be one of the most useful diagnostic techniques for detecting the presence and occurrence of cow mastitis, especially subclinical mastitis. SCC varied with lactation status, age, stress, milking time, milking frequency, season, and udder infection (29, 30). Other cell types such as white blood cells (WBC), macrophages, lymphocytes, and polymorphonuclear leukocytes also play an important role in distinguishing the cow mastitis (31). SCC is the main immune defense mechanism of cow udder, which is composed of neutrophils, monocytes and lymphocytes.

Intramammary infection during mastitis can considerable impact on the mammary gland of dairy cows. The molecular mechanism of cow udder inflammation is very complex because many metabolites involved in the process. Inflammation is main immune defense mechanism of cow's udder or mammary region that can make many types of cells secrete into milk, such as neutrophils, monocytes, macrophages, lymphocytes, and epithelial cells and result in increased SCC, which is related to the inflow of metabolites (such as fat, free amino acids, organic acids, and other low molecular weight metabolites) into milk (24). At present, microorganisms in milk, milk enzymatic reaction, active secretion or leakage in mammary epithelial cells, immune cells, damaged somatic cells, and blood transfer are considered to be related to milk metabolites due to the destruction of blood milk barrier permeability, more blood components leak into milk (24). Some important metabolites, including lactic acid, acetic acid, uracil, isoleucine, butyric acid, and hippuric acid, are related to SCC (32). In addition, mammary epithelial cells, neutrophils, macrophages, and lymphocytes secrete different milk metabolites. For example, neutrophils can secrete several defense compounds and is considered as the first line of defense against bacterial infection (33). The metabolite spectrum of cow mastitis can directly reflect the physiological and health status of udder, which provides a basis for further understanding the overall metabolic mechanism of cow mastitis. Understanding the pathogenesis of cow mastitis will help to prevent and control mastitis, improve welfare, genetic potential, and the profitability of dairy farmers.

### Pathogenesis of Dairy Cow Mastitis

Mitogen activated protein kinases (MAPK) pathway and nuclear factor-κB(NF-κB) pathways are two important inflammatory signal transduction pathways in cow mammary gland cells (34). MAPK is a class of serine threonine protein kinases in cells, including extracellular signal regulated kinases (ERK1/2), c-Jun N-terminal kinases (JNK), p38 MAPK and ERK5. MAPK can react with upstream signals to form a classic MAPK kinase (MAP3K)-MAPK kinase (MAP2K)-MAPK cascade. MAP3K activates the threonine and tyrosine sites of MAP2K and phosphorylates the threonine and tyrosine sites of MAPK when the cell membrane is stimulated. Then MAPK translocates into the nucleus and induces the expression of related inflammatory genes (35). Lipopolysaccharide binding protein (LBP), antigen recognition receptor (CD14), tumor necrosis factor -α (TNF-α) and Interleukin-1β (IL-1β) in milk and blood of dairy cow mastitis were up-regulated, the expression levels of toll like receptor 4 (TLR4), p38 protein kinase and phosphorylated p38 protein (p-p38) induced by inflammation in udder were significantly increased, suggesting that the phosphorylation of related proteins in MAPK signaling pathway may be an important factor in the occurrence of mastitis (36). Additionally, activation of MAPK pathway can also induce the activation of NF-κB pathway and lead to the occurrence of mastitis in dairy cows. Therefore, it is necessary to study the mechanism of MAPK pathway related proteins, which can help us determine the occurrence of mastitis and prevent and control it in time.

Nuclear factor-κB (NF-κB) pathways, including nuclear factors κB inhibitory protein (IκB) kinase complex, IκB, NF-κB transcription factor protein family. When the cell is in a resting state, the P50 and P65 subunits usually form NF-κB dimers and form complexes with IκB, which exist in the cytoplasm in an inactive form. When the cell is stimulated, the IκB kinase complex is activated to phosphorylate the two key serine sites of the IκB protein and then be ubiquitinated by the 26S proteasome. The site of NF-κB is exposed and quickly transferred to the nucleus, and specifically binds with the target gene and promotes the synthesis of TNF-α, IL-1β related protein (37). The mRNA and protein expression of TLR4, NF-κB, IL-1β, and TNF-α were up-regulated in mastitis induced by LPS and activated the TLR4/NF-κB signaling pathway. The activated TLR4/NF-κB signaling pathway will damage the bovine mammary epithelial cells (38). NF-κB is the core regulator of innate immunity. Scientifically, clarifying the regulation mode and mechanism of NF-κB signaling pathway will effectively prevent dairy cow mastitis.

## Metabolomics

Metabolomics is a discipline that studies the collection of all metabolites at a certain time (39), and the research object is mostly small molecular substances with relative molecular mass <1,000 Da (40). A large number of metabolites are analyzed by bioinformatics to find out the different metabolites, and finally clarify the interaction between metabolites through metabolic pathway analysis. Combining the results of genomics, transcriptomics, and proteomics will provide more complete and comprehensive biological spectrum (41). Metabolites can reflect the environment and the nutritional status of cells, the role of drugs and environmental pollutants, and the influence of other external factors. At present, the developed rapidly metabolomics has penetrated into the fields related to human (42) and animal (43), such as disease diagnosis, nutrition (44), food science (45), toxicology (46), and so on. Compared with other omics technologies, metabolomics has the unique advantage that it can reflect the current situation of biological systems, and it is relatively cheaper to implement.

Biological fluid, cell extract, tissue extract and tissue block are the main research objects of metabolomics, while metabolic profile, metabolic fingerprint, and metabolic footprint are the main research methods in metabolomics. Nuclear magnetic resonance (NMR), gas chromatography-mass spectrometry (GC-MS), and liquid chromatography-mass spectrometry (LC-MS) are the most widely used metabolomics techniques with the advantages and disadvantages (Table 1), researchers should choose the appropriate type of metabolome sequencing according to the characteristics of the samples they test. However, there are some disadvantages of metabolomics, firstly, the direct detection of metabolites in biological fluids, tissues and cells still needs to overcome the difficulties of mass spectrometry detection caused by thousands of metabolites in samples, and the further development of metabolomics still needs to improve the detection sensitivity, reduce the background and simplify the sample preparation. Secondly, metabolomics need highly complex data analysis software and more perfect metabolite database to complete the identification of metabolites and network analysis of metabolic pathways (54). In addition, metabolomics studies all the metabolic responses of individuals under the stimulation of exogenous substances, environmental changes, and genetic modification, detects the full picture of this response and its dynamic changes. It is a qualitative and quantitative analysis of all small molecule metabolites in cells of a biological system under limited time and conditions. Therefore, in order to effectively utilize metabolomics under various phases of lactation, variable feeding status, stressors, and other environmental co-factors, we must find the differential metabolites that affect healthy and diseased dairy cows under limited conditions, and choose the real and suitable metabolites that affect the health of dairy cows.

**Table 1:** Comparison of different metabolomics.

**Technology**	**Advantages**	**Disadvantages**	**Detectable metabolites**	**References**
NMR	Non-destructive; Can provide structural information of metabolites; Higher repeatability; Can be recycled for other analysis; Fast; Requires no derivatization and separation; Can be used for metabolite imaging (fMRI or MRS); Compatible with liquids and solids.	Low sensitivity; Cannot be used for qualitative analysis of lipids and other substances; High start-up cost (>US$1 million); Cannot detect or identify salts and inorganic ions; Cannot detect non-protonated compounds.	Sugars, amines, volatile liquids, large molecules (>500 Da), relatively non-reactive compounds, a lower limit of detection of approximately 1-5 μM.	(41, 47–50)
GC-MS	High resolution; Good sensitivity; Detects most organic and some inorganic molecules; Excellent separation reproducibility; Many spectral features can be identified; Compatible with gases and liquids.	The sample needs to be chemically derived; Destructive (sample not recoverable); Requires separation; Cannot be used in imaging; Incompatible with solids; Difficult to identify new compounds.	Silicane, derivatives, Eicosanoids, essential oil, ester, terpene, lipid, paraffin, volatile matter, carotenoid, flavonoid.	(49, 51, 52)
LC-MS	Can separate molecules in liquid phase without derivatization; High sensitivity; Detects most organic and some inorganic molecules; Can be used in metabolite imaging (MALDI or DESI); High measurement range; Compatible with solids and liquids; Can be used in series with quadrupole time of flight (Q-TOF), ion trap time of flight (IT-TOF).	Destructive (sample not recoverable); Higher cost; Slow; Usually requires separation; Poor separation resolution and lower reproducibility versus GC-MS; Not compatible with gases Difficult to identify new compounds; Many spectral features cannot be identified.	Organic acid, organic amine, nucleoside, ionic sample, nucleotide, polyamine.	(51–53)

## Application of Metabolomics in Cow Mastitis

More and more researches on the metabolomics application in milk sample analysis have been carried out with the continuous improvement of metabolomics related instruments and analytical techniques (55). The health status of dairy cows can be detected and the pathological metabolic mechanism of dairy mastitis can be well-revealed by metabolomic sequencing of milk, blood, rumen fluid, and urine of dairy cows. Therefore, more and more experts and scholars use metabolomics to find biomarkers related to cow mastitis (Table 2).

**Table 2:** Research on dairy cow mastitis through metabolomics in various countries.

**Sample type**	**Method**	**Types of mastitis**	**Metabolite**	**Metabolic pathways**	**Country**	**References**
Milk	H-NMR	Clinical mastitis	Acetate, formate, lactate, benzoate, valerate, alanine, histidine, β-hydroxybutyrate, isoleucine, leucine, leucine, threonine valine, N-acetylamino acid, phenylalanine, valerate, alanine, N-acetylglucosamine, hippurate	/	Thailand	(56)
		Subclinical mastitis	N-acetylglucosamine, hippurate, valerate, isoleucine, histidine			
Milk	LC–MS	Clinical mastitis	Thiamine, riboflflavin, xanthine, ceramide acid sphingomyelinase, AIR, L-arginine phosphate	Lipid, vitamin, purine, pyrimidine, amino acid, biosynthetic metabolism of unsaturated fatty acids, steroid hormone biosynthesis	China	(57)
		Subclinical mastitis				
Blood	GC–MS	Subclinical mastitis	Valine, serine, tyrosine, phenylalanine, isoleucine, proline	Propanoate, sphingolipid, protein biosynthesis, ammonia ser recycling, val, leu, Ile degradation, porphyrin, bile acid synthesis, parturition ammonia, methionine, recycling, aspartate, glutathione, parturition propanoate	Canada	(58)
Urinary	DI/LC-MS	Subclinical mastitis	C3:1, C3-OH, C5-DC (C6-OH), C5-M-DC; C14:1, C18:2; PC aa C34:2, PC ae C42:1, Asp, carnosine, glu, his, SDMA, thr, 12, C14, C16:2, PC ae C36:4	Sphingolipid, selenoamino acid, methionine, cys, fatty acids biosynthesis, asp homocysteine degradation, tyr, ammonia recycling, phosphatidylethanolamine biosynthesis, arachidonic acid, malate-aspartate shuttle	Canada	(59)
Milk	LC-MS	Mastitis	Taurochenodeoxycholic acid, lactose, taurocholic acid, glycodeoxycholate, glycocholate, cholate, hippurate	ala, asp, glu, purine, pyrimidine, eicosanoids, ascorbate, aldarate	UK	(60)
Milk	GC-TOFMS	Subclinical mastitis	Phenylpyruvic acid, homogentisic acid, xanthine, uridine, glycerol	Galactose, pentose, glucuronate interconversion, TCA, ala, D-glutamine, biosynthesis pathways, D-glutamate, asp, glu, arg, gentamicin, biosynthesis, neomycin, sucrose, starch, kanamycin,	China	(61)
Milk	NMR	Mastitis	Lactate, butyrate, isoleucine, acetate, β-hydroxybutyrate, hippurate, fumarate	/	Denmark	(62)
Milk, rumen liquid	LC-MS	Clinical mastitis	12-oxo-20-dihydroxy-leukotriene B4, d 10beta - hydroxy - 6beta - isobutyrylfuranoeremophilane, 2-phenylbutyric acid	Limonene, pinene degradation, arachidonic acid, butanoate	China	(63)
		Subclinical mastitis	Methenamine, 6-methoxymellein, 5-HMF	Biosynthesis of plant secondary, furfural degradation, formaldehyde biosynthesis		
Plasma, milk	NMR	Clinical mastitis	Ketone bodies (3-hydroxybutyrate and acetoacetate), short-chain fatty acids, Citrate, lactose	/	Swedish	(64)
Milk	UPLC-Q-TOF MS^E^	Clinical mastitis	d-glycerol-1-phosphate, glucose, arginine, Leu-Leu, citrate, hippurate, sn-glycero-3-phosphocholine, lcarnitine, 4-hydroxyphenyllactate,	Galactose, glycerophospholipid, carnitine, tyrosine, phenylalanine, krebs circle, AA/peptides	China	(65)
		Subclinical mastitis	d-glycerol-1-phosphate, cis-aconitate, benzoic acid, arginine, l-carnitine, Leu-Leu			
Milk	UPLC-Q TOF-MS	Subclinical mastitis	VB2, malic acid, 3-indoleacrylicacid, tyr-cys, cytosine, palmitoyl-l-carnitine, l-sodium stearoyl glycine, VB2, VB5, glucose, lactose, arachidonic acid, L-Carnitine, stearic acid, benzoylglycine, phenylalanine	Lipid, amino acid, peptides, glucose, palmidrol, N-Acetyl-5-oxonorvaline, spermine, ethyl cinnamate, ethyl hexanoate, palmitic acid, fructose 6-phosphate	China	(66)
Milk	UPLC-QTOF-MS^E^	Clinical mastitis	D-Glyceraldehyde 1-phosphate, glucose, citric acid, aconitic acid, 4-Hydroxyphenyllactic acid, lactose, benzoylglycine, phosphorylcholine, malic acid, L-Carnitine	Glycerophospholipid, phenylalanine, TCA, tyrosine, carnitine, peptides, arg, nucleic acid, vitamin, secondary metabolim	China	(67)
		Subclinical mastitis	Short chain polypeptide, L-Carnitine, aconitic acid, pro, arg malonylcarnitine, D-Glyceraldehyde 3-phosphate, benzoic acid, val, lie			
Milk	GC–MS	Clinical mastitis	Sulfifides, ketones, amines, acids, ethanol, acetic acid, trimethylamine, 2-pentanone, 2,3-butanedione, 2-methylbutanal, methane-thiol, dimethylsulfifide	/	Sweden	(68)

### Milk Metabonomics Analysis of Cow Mastitis

Milk is the secretion of udder. Milk components can reflect the metabolic state of udder, so it can be used to infer the systems biology of the mammary gland cells in different physiological and pathological conditions, and often used to detect mastitis in cows. The potential marker metabolites discovered in milk mastitis metabolomics are mainly involved in linoleic acid metabolism, glycerophospholipid metabolism, and amino acid metabolism metabolic pathways. A total of six different metabolites were found in whey samples of mastitis, which were lactic acid, alanine, pyruvate, succinic acid, sn-glycerin-3-phosphate choline, formic acid (62, 69). The level of lactic acid, pyruvate, succinic acid, formic acid, and sn-glycerol-3-phosphocholine metabolism increases with somatic cells increases, the level of alanine metabolism decreases. The presence of bacteria in milk produces a unique metabolic fingerprint and means an increasing in the concentration of lactic acid. The increasing of lactic acid level indicates the increasing of bacterial metabolic level (62). Lactic acid concentration increased 10 or 30 times in whey samples with subclinical or clinical mastitis, respectively (70), and the increasing of lactic acid may be caused by leukocyte metabolism. The concentration of lactic acid will increase significantly within 24 h after infection (70). The rapid response of lactic acid to mammary gland infections makes it a potential and very useful biomarker for diagnosing mastitis in dairy cows.

With the increasing of anaerobic respiration and anaerobic glycolysis of glucose, the metabolic levels of lactic acid and pyruvate in whey increased (71). Lactate dehydrogenase, a non-lysosomal hydrolase, can be released from neutrophils, macrophages, damaged mammary epithelial cells and stromal cells in the process of cow udder inflammation. Lactic acid dehydrogenase can convert lactic acid into pyruvate (72). Pyruvate is the starting point of gluconeogenesis and the end product of glycolysis, it can also be produced by alanine aminotransferase (73). Pyruvate, an important intermediate metabolite, which can be produced by succinic acid pathway or lactic acid pathway (74). Pyruvate dehydrogenase complex can help pyruvate to convert the acetyl coenzyme A and enter the tricarboxylic acid (TCA) cycle (75).

TCA cycle plays an important role in cell respiration and energy supply to all living cells (76). The increasing of pyruvate metabolism level may lead to the increasing of alanine transamination. The increasing of alanine metabolism level may be the result of the increasing of pyruvate metabolism level (77). However, the differences of pyruvate metabolism between healthy cows and cow mastitis need to be further verified by large-scale sample experiments. The metabolic level of succinic acid in whey of mastitis increased, which is contrary to the research results of Thomas (78). Succinic acid is an important metabolite related to TCA cycle and an important starting material and intermediate product of TCA cycle (79). TCA cycle is an important link in the metabolic pathways of sugar, lipids, and amino acids. TCA will have a corresponding impact on other metabolic pathways if there is any problem in this process. Therefore, the change of succinic acid metabolism level will inevitably disturb TCA cycle and have a certain impact on the body of dairy cows. Succinic acid metabolism in cow mastitis is rarely reported, but the succinic acid metabolism of dairy cow mastitis will help us to understand the relationship between mastitis and TCA cycle, and solve the incidence of dairy cow mastitis.

The metabolic level of sn-glycerin-3-phosphate choline was decreased in cow mastitis. Phosphatidylcholine is a phospholipid component that is essential to the integrity of cell membrane structure. The increasing of sn-glycerin-3-phosphate choline metabolic level is an important manifestation of cell injury or pathological changes (inflammation or necrosis) (80). The content of glycerophospholipids in milk decreased with the infection process of cow mastitis, which may be due to the destruction of cell membrane or hydrolysis by carboxylesterase and phospholipase during mastitis (81). Formic acid may be the single carbon source of N5, N10-methyl-TNF, which is an irreplaceable and crucial substance in the biosynthesis of nucleic acids (82). A large amount of formic acid is used to treat inflammatory diseases involved in purine nucleotide biosynthesis (83). Dairy cow may perceive the inflammation when udder was infection, and formic acid is often used to treat the inflammation caused by pathogenesis. However, a large number of samples are needed for research to support this finding, which may provide new ideas for future research on the pathogenesis of mastitis.

In addition, different metabolites in milk can also indirectly reflect whether cows have mastitis or not. The contents of butyric acid, isoleucine, glycerol, peptides, acetic acid, and β-hydroxybutyric acid in milk with high SCC (>7.2 × 10^5^/mL) increased significantly (65), while the contents of lactose, hippuric acid, and fumaric acid decreased. Therefore, butyric acid, β-hydroxybutyric acid, isoleucine, hippuric acid, fumaric acid, glycerol, peptides, and fumaric acid can be used as the new markers in milk with high SCC, and high SCC is also a typical feature of subclinical mastitis and clinical mastitis, so these metabolites can indirectly reflect whether cows have mastitis or not (62). The bile acid nuclear receptor FXR signaling pathway is significantly up-regulated in mastitis milk. It is suggested that bile acids may be participate in the inflammatory response of the udder (60).

### Blood Metabonomics Analysis of Cow Mastitis

Blood is easy to collect and can completely reflect the physiological and biochemical state of dairy cow in time. So it is often used to detect the occurrence of various diseases in cows. The metabolomics of amino acids, carbohydrates, and lipids in milk with subclinical mastitis during perinatal period were analyzed by GC-MS, valine, serine, tyrosine, and phenylalanine could be used as the markers of subclinical mastitis in 4–8 weeks before delivery, while valine, isoleucine, serine, and proline can be used as the markers of subclinical mastitis in 4–8 weeks post-partum (58). Therefore, the metabolic changes of amino acids can be used to predict the risk of subclinical mastitis.

Six different metabolites were found in plasma samples with mastitis, which were malonic acid, phenylalanine, isoleucine, leucine, glycine, and glucose. Malonic acid is a competitive inhibitor, which plays an important role in the respiratory electron transport chain of the TCA cycle (84). The increasing of malonic acid metabolism level may be the reason of the disorder of TCA cycle metabolism in dairy cows. Malonic acid may be a new marker for the early diagnosis of cow clinical mastitis, and provide a new research direction for researchers. Phenylalanine needs to maintain enough tetrahydrobiopterin and produce nitric oxide through nitric oxide synthase in activated macrophages and other leukocytes. In addition, phenylalanine is also a precursor of tyrosine (85). Lack of phenylalanine and tyrosine can damage the immune response of chickens, which can be reversed by adding alanine and tyrosine to the diet (85). The decreasing of phenylalanine and other metabolites in the blood with mastitis will lead to the decreasing of the production of neurotransmitters (86) and the overall disorder of the endocrine system.

The metabolism levels of isoleucine and leucine in blood with mastitis were decreased. The metabolic processes of branched chain amino acids such as isoleucine and leucine are almost the same. They are essential amino acids for the cow, and they are involved in glucose metabolism by fatty acids β-oxidative pathway to produce succinyl coenzyme A and acetyl coenzyme A, which ultimately meet the body's energy needs (87). Branched chain amino acids will play an important role in the energy supply and can realize the continuous energy supply to the body by strengthening the function of gluconeogenesis when negative energy balance (NEB) occurs (88). Isoleucine and leucine will directly affect the TCA cycle and the energy supply of glycolysis pathway when the metabolism level of isoleucine and leucine decreases, which suggest that the important intermediate links of TCA cycle and glycolysis pathway are disturbed in mildly clinical mastitis (89). Isoleucine is highly correlated with SCC level in cow mastitis (62). The increase of amino acids may be related to the higher activity of pathogen specific fermentation and protein degradation. Therefore, isoleucine and leucine have potential to be used as biomarkers for the diagnosis of cow mastitis, but further experimental verification is still needed.

Glycine, a glycogenic amino acid, is one of the main sources of cell energy in the body (90). The low level of glycine metabolism in blood of cow mastitis indicate the lack of energy substrate (85). Glycine usually is participate in glucose metabolism as a glycogenic substance under the condition of severe energy deficiency (91). At present, there are few reports about the differential metabolic expression of glycine in dairy cow mastitis. Further experimental research on glycine metabolism differential expression need to be done in the future, which may provide a new idea for the early diagnosis of cow mastitis.

The concentration of glucose in the blood with clinical mastitis will decrease (60). Although the exact reason for this change is unclear, one possible explanation is that blood flows to the infected udder and result in a limited supply of blood sugar secreted by the cells (92). Another hypothesis is that glucose is transported from the milk to the extracellular pathway to maintain the osmotic balance between the extracellular environment and the milk with the increase of Na^+^ and Cl^−^ when the body suffers from mastitis. In addition, hypoglycemia may be due to the accumulation of cells at the site of infection, which depletes the glucose in the local area. The appropriate level of blood glucose metabolism is essential for maintaining milk secretion (93). Therefore, the decreasing of blood glucose level can lead to the occurrence of mastitis with the stop of milk secretion and the decrease of milk production. In conclusion, metabolomics can be used to distinguish the different metabolites in blood with mastitis, which is helpful for the early diagnosis and pathological research of dairy cows clinical mastitis.

### Rumen Microbiome Metabonomics Analysis of Cow Mastitis

The rumen is considered as one of the major organs in dairy cows, which directly affect milk production and health of the host. Recent studies have shown that gastrointestinal microbiota play a vital role in inflammation of extra-intestinal tissue, such as mammary gland (94). Intestinal bacteria may be derived from milk, and in turn affect the microorganism in the udder after the formation of intestinal flora (95). Therefore, metabolomics research on rumen microorganisms will help us to find more comprehensive markers that affect dairy cow mastitis.

A study on rumen microbes with cow mastitis revealed that the infected degree of udder. The concentrations of lactic acid, acetate, propionate, butyrate, valerate, and total volatile fatty acids in rumen were significantly decreased (63). Bacteria associated with intestinal inflammation and oral inflammation are significantly increased in the rumen of dairy cows with clinical mastitis. The ruminiclostridium_9 and enterorhabdus were increased with the increasing of methenamine, 5-hydroxymethyl-2- furancarboxaldehyde (5-HMF) and 6-methoxymellein in the rumen of subclinical mastitis (63). In addition, the bacteria and probiotics produced by short-chain fatty acids (SCFAs) in rumen were significantly reduced with decreasing of 2- phenylbutyric acid (2-PBA) (63). Zhong et al. (96) compared the rumen changes of high and low SCC in cow and found that cows with higher SCC (3 × 10^6^/mL) had lower milk yield, poorer milk composition content, reduced rumen VFA concentrations and higher rumen bacterial diversity. The relative abundance of phyla SR1 and actinobacteria, unclassified family clostridiales and genus butyrivibrio were significantly different among cows with different SCC levels. To sum up, we can detect cow mastitis by decreasing of lactic acid, butyric acid, propionic acid, acetic acid, valeric acid, short-chain fatty acids (SCFAs)-producing bacteria and probiotics in the rumen. However, we need to find the exact time points of these substances change according to the spatiotemporal nature of metabolomics. In addition, the correlation among the changes in rumen microorganisms and metabolites and the udder health of dairy cows is still unclear, and the mechanism of the relationships between mastitis and ruminal internal environment still need further investigation.

### Urinary Metabonomics Analysis of Cow Mastitis

Urine is one of the important substances for detecting the health status of dairy cows. In the daily test of dairy cow urine, the possible disease risk of dairy cows can be judged by indicators such as WBC, pH value, ketone body, and so on (97). In addition, urine is also a body fluid that can be easily collected and can be used to monitor cows for subclinical mastitis during the dry period as well as during the first 2 months after parturition. A urine pen-side test would be very desirable to avoid more time-consuming measurement of SCC at specialized laboratories. The milk and blood samples have been used in dairy cow mastitis metabolomics research. However, Gz et al. (59) found that metabolites related to lipid, carbohydrate, and amino acid pathways have changed in the urine of pre-subclinical mastitis, subclinical mastitis, and post-subclinical mastitis cows. A total of 14 urinary metabolites were identified and measured as during the −8 and −4 w pre-partum and during the week of disease diagnosis, those metabolites play important roles in distinguishing the healthy and cow mastitis (59). Additionally, seven metabolites were found after subclinical mastitis diagnosis at +4 and + 8 wks post-partum. There are few studies on the detection of mastitis by urine, but many indicators can be detected in urine. Therefore, urine is very important for the clinical diagnosis, judgment and prevention of many diseases. A large number of studies on urine detection of dairy cow mastitis are necessary in the future. In addition, accuracy determination of these metabolites in identifying mastitis requires further verification and more research is warranted to elucidate the reason why metabolites are excreted in pre-subclinical mastitis and subclinical mastitis cows by lipid, carbohydrate and amino acid pathways.

## Conclusion

Mastitis is the key factor to restrict the health and safety of dairy cows. Omics technology can find the expression changes of metabolites in milk and blood samples of dairy cows, which can not only further reveal the pathogenesis of mastitis and find the specific markers of mastitis, but also enrich the methods of prevention and treatment of mastitis. Metabolomics can detect biological fluids (such as urine, blood, milk, rumen), tissues, feces, cells, or culture medium. Blood, milk, and urine are the most easily obtained body fluid samples, especially blood with a lot of metabolic information and good stability can be used as biological samples in animal metabolomics research. In addition, collecting blood, urine, and milk samples is less harmful to animals and conducive to the development of non-invasive diagnosis and treatment technology by using the results of metabolomics detection, and can also be used for the screening, validation and clinical transformation of biomarkers. It is believed that omics technology will provide a new direction for clinical diagnosis and treatment of cow mastitis with the continuous maturity of omics technology in the follow-up stage.

## Prospects

As the most downstream of gene expression, metabolomics can amplify the small changes in gene and protein expression at the metabolite level, and can more fully reflect cell functions. The application of metabolomics technology in the dairy cow mastitis can analyze differential metabolites and identify related biomarkers, and then reveal the physiological and pathological changes of dairy mammary glands. There are some disadvantages in metabolomics, the composition of metabolites is complex, many substances cannot be detected due to the limitations of the current method or the low sample content, and the processing before metabolome sequencing will lost some metabolites and result in analyze error. Metabolomics research with very small sample size requires the development of ultra-sensitive trace metabolite detection methods to achieve a wide coverage of metabolites. Therefore, all the above defects of metabolomics need to be improved in the future.

Although single metabolomics technology can detect abundant metabolites, it also has certain limitations. Therefore, it may be better to integrate metabolomics data with genomics, transcriptomics, and proteomics data to establish a complete database, which can help us to more deeply reveal the occurrence mechanism, action mechanism, and pathogen defense mechanism of mastitis. But how to carry out the most effective data and information processing on these big data is still a technical bottleneck in omics research. In addition, the occurrence of dairy cow mastitis is spatio-temporal and will gradually increase or decrease over time and produce many differences metabolites at different times. Most of the volatile metabolites are products of different mastitis pathogens, and the metabolites cannot be completely distinguished. Besides, metabolomics is also affected by internal and external environments, nutrition, management, and various stressors. In summary, accurate and efficient qualitative analysis, quantitative analysis, and clinical verification of metabolites in a specific physiological period can clarify the relevant metabolic pathways, and those metabolites can be used as the specific and sensitive marker for mastitis.

For the prediction of mastitis susceptibility of healthy Holstein cows, we must first monitor some behavioral changes of healthy cows and mastitis cows, and develop an automatic health monitoring system that can record physical parameters. Different metabolites in milk samples and blood samples of different stages of healthy and mastitis cows are detected by metabolomics, so as to distinguish the different metabolites. Use the MIR spectral data obtained from monthly milk records to predict the metabolites that appear in dairy cows in the early stages of illness and find metabolites that can reflect mastitis. Combining cow behavior information (heart rate, blood oxygen, body temperature, rumination, and physical activity), production information (lactating days, parity, milk production, and SCC), feeding conditions and environment, establish a MIR-based prediction model of mastitis milk metabolites, and determine the differences in the distribution of metabolites and possible pathways. According to mastitis metabolites and possible metabolic pathways for timely prevention and treatment.

## Author Contributions

HH and ZF were writing—original draft preparation, investigation, and validation. TM, ZW, and YuM were revision and supervision. YaM was conceptualization. All authors contributed to the article and approved the submitted version.

## Funding

This research was supported by grants from National Natural Science Foundation of China (No. 32060765), Science and Technology Planning project of Inner Mongolia Autonomous Region (No. 2021GG0025), Ningxia University Scientific Research Start-up Project (No. 030900002154), Modern Agro-industry Technology Research System (CARS-36), and Ningxia Hui Autonomous Region Key R&D Projects (No. 2021BEF01001).

## Conflict of Interest

The authors declare that the research was conducted in the absence of any commercial or financial relationships that could be construed as a potential conflict of interest.

## Publisher's Note

All claims expressed in this article are solely those of the authors and do not necessarily represent those of their affiliated organizations, or those of the publisher, the editors and the reviewers. Any product that may be evaluated in this article, or claim that may be made by its manufacturer, is not guaranteed or endorsed by the publisher.
